# TRIPLE COMBINATION OF HYDROQUINONE, TRETINOIN AND MOMETASONE FUROATE WITH GLYCOLIC ACID PEELS IN MELASMA

**DOI:** 10.4103/0019-5154.49005

**Published:** 2009

**Authors:** Kiran V Godse

**Affiliations:** *From the Shree Skin Centre, 22 L Market, Sector 8, Nerul, Navi Mumbai-400 706, India. E-mail: drogodse@yahoo.co.in*

Sir,

Melasma is a very common condition that develops exclusively on the exposed areas of the face. The basic mechanisms involved in the pathogenesis of this difficult to treat disorder is still not very well known. Despite the large number of depigmenting agents available, the treatment of melasma is often unsuccessful and disappointing and is still a challenge for dermatologists.

We studied triple combination of 4% hydroquinone, 0.05% Tretinoin and 0.1% mometasone furoate cream with glycolic acid peels, in the treatment of melasma.

Ten patients with melasma on the face (two males and eight females), in the age group of 21-50 (mean age 27), with duration ranging from three months to seven years, were included in the study. Those with a history of recurrent herpes, and nursing and pregnant women were not included in the study. All the patients were informed about the procedure and their consent was taken. All patients with melasma were put on a maintenance regimen of Tretinoin (0.05%), Hydroquinone (4%) and mometasone furoate (0.1%), to be applied at night. The patients were instructed to apply medication all over the face, after mixing in an equal proportion on the palm. In the morning, physical sunscreen of SPF 19 (Micronized zinc oxide 15%) was used. Serial Glycolic acid peels were used at three weekly intervals, for two to six minutes, depending on tolerance and erythema. Glycolic acid of 57% with free acid of 55% and pH of 2.3 was used in gradually increasing duration of application on the face. Post peel care was in the form of calamine lotion and sunscreens.

The patients were instructed to use mometasone furoate on alternate nights, after six weeks of application, to avoid corticosteroid side effects. All the patients were photographed before starting the therapy and at the end of four peels.

Melanin pigment intensity scale was rated as a 3-grade scale, in relation to the patient's normal facial skin. Grade I: slightly more pigmented, Grade II: moderately more pigmented, Grade III: markedly more pigmented. The overall response to the treatment was rated by the physician and the patient, at end of the study as - excellent (75-100% improvement), good (50-74% improvement), fair (20-49% improvement), and poor (0-19% improvement). Adverse effects were noted during each visit.

The results were noted after four peels, in the form of more than 50% improvement in pigmentation in five out of 10 patients. Two patients showed more than 75% improvement. The remaining three patients showed fair improvement. Adverse reactions like hyperpigmentation and irritation were encountered in one patient. Persistent erythema was noted in one patient.

Hydroquinone remains the most prescribed bleaching agent worldwide, and is still the gold standard for treatment of hyperpigmentation, although since 2001 its use has been banned in Europe as an ingredient in cosmetics. Tretinoin acts on different levels: it facilitates the removal of pigment by accelerating the keratinocytes' turnover; it enhances hydroquinone penetration through the stratum corneum; and, it protects hydroquinone from oxidation. The introduction of a corticosteroid is aimed to reduce inflammation as a side effect of both hydroquinone and tretinoin, but it also inhibits melanocyte metabolism.

The US Food and Drug Administration has approved a modified combination of the Kligman formulation, containing 4% hydroquinone, 0.05% tretinoin, and 0.01% fluocinolone acetonide. This association has proved its efficacy without significant side effects, in a multicentric safety study performed over a 12-month period on 569 subjects affected with moderate to severe melasma. By month twelve, the melasma was resolved or almost resolved in 80% of the subjects.[1] In a recent comprehensive review of the long-term and short-term treatment of melasma with a once-daily application of the triple combination cream, Torok[2] reports that the results of the numerous extensive trials highlight both the efficacy and the safety of the treatment, with the adverse events being mild and rare. A recent study has shown that other than their capacity to increase cellular epidermal turnover, which leads to pigment dispersion, these hydroxyacids directly inhibit tyrosinase activity.[3]

Serial Glycolic acid (GA) peels enhance the efficacy of topical regimen with more rapid initial response and overall lightening of skin. This regimen is useful in recalcitrant cases and when rapid response is desired. A 50 to 70% Glycolic acid has proven to be effective and safe, even in subjects with ethnic skin, as highlighted by a study performed on 25 Indian women affected by melasma, with a minimum MASI of 15. A 50% Glycolic acid peeling was performed once a month, for three months. Significant reduction of MASI was achieved in 91% of the subjects; epidermal forms responded better than mixed ones, dermal type did not respond at all.[4] Forty Indian patients with Fitzpatrick skin types III to IV, with moderate to severe melasma, were randomized to treatment with the modified Kligman formulation daily plus 6 serial Glycolic Acid treatments at 3-week intervals or to modified Kligman formulation alone. The group receiving GA showed a trend toward more rapid and greater improvements than the group receiving only the modified Kligman formulation. Adverse events were minimal in both groups.[5]

Mometasone furoate is a newer potent glucocorticoid agent, ideally suited for aggressive topical treatment of severe localized dermatoses. Being more lipophilic, mometasone also may remain topically in the skin, in high concentrations with lesser systemic absorption, and, in turn, produce lesser incidence of systemic side effects. The special base and excipients used in the formulation of mometasone may also have an enhancing effect on its overall superior clinical efficacy.[6]

We found this triple combination suitable for Indian skin. The addition of Glycolic acid peels enhances the overall improvement in melasma. The treating dermatologist must be constantly aware of the psychosocial impact of pigmentary imperfections, and he/she must attempt to use available therapies and give the best to the patient.
